# LncRNA PVT1 regulates atrial fibrosis via miR-128-3p-SP1-TGF-β1-Smad axis in atrial fibrillation

**DOI:** 10.1186/s10020-019-0074-5

**Published:** 2019-03-20

**Authors:** Feng Cao, Zhe Li, Wen-mao Ding, Ling Yan, Qing-yan Zhao

**Affiliations:** 1 Department of Cardiology, Renmin Hospital of Wuhan University, NO.99 Zhangzhidong Road, Wuchang District, Wuhan, 430060 China; 2 Cardiovascular Research Institute, Wuhan University, Wuchang District, Wuhan, 430072 China; 3 Hubei Key Laboratory of Cardiology, Wuhan, 430060 China

**Keywords:** PVT1, miR-128-3p, Sp1, TGF-β1/Smad signaling, Atrial fibrosis, Atrial fibrillation

## Abstract

**Background:**

Long non-coding RNAs (lncRNA) plasmacytoma variant translocation 1 (PVT1) has been shown to be associated with liver fibrosis. Nevertheless, the role of PVT1 in atrial fibrosis remains undefined. This study aims to elucidate the pathophysiological role of lncRNA PVT1 in the regulation of atrial fibrosis and to explore the underlying mechanism.

**Methods:**

Expression of PVT1, miR-128-sp, and Sp1 were examined in human atrial muscle tissues and angiotensin-II (Ang-II)-induced human atrial fibroblasts. Furthermore, the role of PVT1 in regulating atrial fibrosis in Ang-II-treated human atrial fibroblasts and Ang-II-induced atrial fibrosis in mice was investigated. Moreover, the interaction among PVT1, miR-128-3p, and Sp1 were examined using bioinformatics, expression correlation analysis, gain- or loss-of-function assays, RIP assays, and luciferase reporter assays. The involvement of transforming growth factor beta 1 (TGF-β1)/Smad pathway in this process was also explored.

**Results:**

PVT1 was increased in atrial muscle tissues from AF patients and positively with collagen I and collagen III. In vitro assay revealed that PVT1 overexpression facilitated the Ang-II-induced atrial fibroblasts proliferation, collagen production, and TGF-β1/Smad signaling activation, whereas PVT1 knockdown caused the opposite effect. In vivo assay further confirmed that PVT1 knockdown attenuated the Ang-II-induced mouse atrial fibrosis. Mechanically, PVT1 acted as a sponge for miR-128-3p to facilitate Sp1 expression, thereby activating the TGF-β1/Smad signaling pathway.

**Conclusion:**

LncRNA PVT1 promotes atrial fibrosis via miR-128-3p-SP1-TGF-β1-Smad axis in atrial fibrillation.

**Electronic supplementary material:**

The online version of this article (10.1186/s10020-019-0074-5) contains supplementary material, which is available to authorized users.

## Background

Atrial fibrillation (AF) is one of the most common arrhythmias in the clinic and are responsible for important population morbidity and mortality (Nattel and Harada 2014). The efficacy of presently available therapeutic approaches is limited. Thus, elucidating the mechanism underlying AF development is essential for AF therapeutic innovation.

Atrial fibrosis is the hallmark of atrial structural remodeling in AF and has emerged as an important pathophysiological contributor to AF (Nattel 2017). Atrial fibrosis is characterized by abnormal proliferation of atrial fibroblasts and excessive deposition of extracellular matrix (ECM) (Wang et al. 2017). Atrial fibrosis influences AF development by transforming growth factor beta 1 (TGF-β1)/Smad pathway (Wang et al. 2018). TGF-β1 plays an important role in the development of atrial fibrosis (Sun et al. 2015; Choi et al. 2012). The tgf-β1 expression can be induced by angiotensin-II (Ang-II) that can stimulate fibroblasts proliferation and ECM production and then contribute to atrial fibrosis (Dobaczewski et al. 2011; Su and Zhang 2018).

Long non-coding RNAs (lncRNAs) are a class of transcripts longer than 200 nt in length and involved in multiple biological processes. However, to date, only a small number of lncRNAs, such as lncRNA H19 (Tao et al. 2016) and lncRNA MIAT (myocardial infarction-associated transcript) (Qu et al. 2017), have been identified associated with cardiac fibrosis. The lncRNA PVT1 (plasmacytoma variant translocation 1) has been reported to be up-regulated in a variety of malignancies and can promote tumor cell proliferation, migration and tumor growth and metastasis (Cui et al. 2016; Tian et al. 2019). A previous study indicated that PVT1 is highly expressed in fibrotic liver tissues, and knockdown of PVT1 can attenuate collagen deposition and liver fibrosis (Zheng et al. 2016). However, the role of PVT1 in other tissues fibrosis, including atrial fibrosis, remains undefined.

Our bioinformatics analysis revealed that PVT1 harbors predictive binding sites for miR-128-3p. Furthermore, miR-128 is capable of targeting the transcription factor specificity protein 1 (Sp1) (Dai et al. 2016) that binds to TGF-β1 and can activate TGF-β1 expression (Martin-Gallausiaux et al. 2018). It has been well-known that lncRNAs can exert certain roles by acting as a competitive endogenous RNA (ceRNA) to segregate miRNAs away from target mRNAs (Xiong et al. 2018; Wei et al. 2018). Therefore, we hypothesized that PVT1 might act as a ceRNA by sponging miR-128-3p to abolish the inhibitory effect of miR-128-3p on Sp1, thereby activating the TGF-β1/Smad signaling pathway and then promoting atrial fibrosis. To address this, we detected PVT1 expression in human atrial muscle tissues and Ang-II-induced human atrial fibroblasts. Furthermore, we investigated the interaction between PVT1, miR-128-3p, and Sp1. Moreover, we also explored the role of the PVT1*/*miR-128-3p/Sp1 axis in regulating atrial fibrosis in AF.

## Materials and methods

### Patients and tissue samples

Patients who underwent heart valve replacement surgery were selected from Renmin Hospital of Wuhan University. Their general clinical data such as age, sex, cardiac function (NYHA) classification and left ventricular ejection fraction (LVEF) were collected (Table 1). Patients with previous coronary atherosclerotic heart disease, chronic pulmonary heart disease, infective endocarditis, hyperthyroidism, severe dysfunction of liver and kidney, and malignant tumors were excluded. They were divided into the sinus rhythm (SR) group (*n* = 20) and the atrial fibrillation (AF) group (*n* = 30) and their atrial muscle tissues were collected and stored in liquid nitrogen for subsequent experiments.

**Table 1:** General clinical data of the patients in the two groups (mean ± SD)

Parameter	SR (*n* = 20)	AF (*n* = 30)
Age	51 ± 11	48 ± 8
Male/female	10/10	14/16
cardiac function (NYHA) classification	I~II: *n* = 2;	I~II: *n* = 3;
	III: *n* = 15;	III: *n* = 23;
	IV: *n* = 3;	IV: *n* = 4;
SBP (mm Hg)	114.21 ± 12.25	121.52 ± 12.28
DBP (mm Hg)	71.53 ± 8.99	76.89 ± 12.15
LAD (cm)	3.22 ± 0.21	4.45 ± 0.34^*^
RAD (cm)	3.55 ± 0.32	3.72 ± 0.39
LVEF (%)	57.65 ± 3.13	55.18 ± 3.46

*SR* sinus rhythm, *AF* atrial fibrillation, *SBP* systolic blood pressure, *DBP* diastolic blood pressure, *NYHA* New York Heart Association, *LAD* left atrium diameter, *RAD* right atrial diameter, *LVEF* left ventricular ejection fraction; ^*^P < 0.05 vs. SR

The protocol was approved by the Ethics Committee of the Renmin Hospital of Wuhan University. Informed written consent was obtained from all participants.

### RNA isolation and quantitative real-time reverse transcription PCR (qRT-PCR)

Briefly, total RNA was extracted from atrial muscle tissues or human atrial fibroblasts using TRIzol reagent (Invitrogen, Thermo Fisher Scientific, Inc., Waltham, MA, USA) according to the manufacturer’s protocol. RNA was then reverse transcribed into cDNAs using the Reverse Transcription System Kit (Takara, Dalian, China). The cDNA templates were amplified by qRT-PCR using SYBR Green PCR Mix (TaKaRa) with the following primers (Zhou et al. 2016; Nakajima et al. 2016; Wu et al. 2017; Lee et al. 2012; Huang et al. 2015): PVT1-F, 5′- TGAGAACTGTCCTTACGTGACC -3′, PVT1-R, 5′- AGAGCACCAAGACTGGCTCT -3′; Collagen I-F, 5′- CTGGTCCCCAAGGCTTCCAAGGTC -3′, Collagen I-R, 5′- CCATCATTTCCACGAGCACCAGCA -3′; Collagen III-F, 5′- GGTCCTCCTGGAACTGCCGGA -3′, Collagen III-R, 5′- GAGGACCTTGAGCACCAGCGTGT -3′; miR-128-3p-F, 5′- -GGTCACAGTGAACCGGTC -3′, miR-128-3p-R, 5′- GTGCAGGGTCCGAGGT-3′; Sp1-F, 5′- ATGCCTAATATTCAGTATCAAGTA -3′, Sp1-R, 5′- CCCTGAGGTGACAGGCTGTGA -3′; GAPDH-F, 5′- TGTTCGTCATGGGTGTGAAC -3′, GAPDH-R, 5′- ATGGCATGGACTGTGGTCAT -3′; U6-F, 5′- CTCGCTTCGGCAGCACA -3′, 5′- AACGCTTCACGAATTTGCGT -3′. Comparative quantification was determined using the 2^-ΔΔCt^ method. Expression of PVT1 and miR-128-5p was normalized to U6 small nuclear RNA. Expression of Collagen I, Collagen III, and Sp1 was normalized to GAPDH.

### Western blot analysis

Briefly, total protein was extracted from atrial muscle tissues of the patients, human atrial fibroblasts, or mouse atrial muscle tissues using a RIPA lysis buffer kit (Santa Cruz Biotechnology, Inc., Dallas, TX, USA). Protein lysates were separated on 10% SDS-PAGE and transferred to polyvinylidene difluoride (PVDF) membranes (Millipore Corp. Billerica, MA, USA). After being blocked in 5% fat-free milk overnight at 4 °C, the membranes were then incubated with the following primary antibodies: Collagen I (1:1000; Abcam, Cambridge, MA, USA), Collagen III (1:1000; Abcam), TGF-β1 (1:1000; Abcam), Smad2 (1:1000; Cell Signaling Technology Inc.), Smad3 (1:1000; Cell Signaling Technology Inc.), and Sp1 (1:1000; Cell Signaling Technology Inc.) at 4 °C overnight. Then the membranes were incubated with the secondary antibody IgG at room temperature for 1 h. The band intensity was quantified with software Quantity One. β-actin served as the loading control.

### Immunohistochemistry

Immunohistochemistry (IHC) was performed to measure Collagen I expression in human atrial muscle tissues. Briefly, the 4-μm thickness sections were deparaffinized in xylene and hydrated using an ethanol-deionized water series. Afterwards, sections were treated with 3% H_2_O_2_ in methanol to block endogenous peroxidase activity. After being blocked with 30% goat serum, sections were incubated with the anti-Collagen I antibody (1: 500; MAB1819; R&D Systems, USA) at 37 °C for 1 h, followed by the biotin-labeled secondary antibody IgG at 37 °C for 20 min. The sections were stained with DAB and the Collagen I-positive signal was recognized when brownish-yellow granules were present. The sections were then counterstained with hematoxylin and observed under an Olympus BH-2 light microscope (Nikon Corporation, Tokyo, Japan).

### Isolation and identification of human atrial fibroblasts

Human atrial fibroblasts were cultured by the tissue block adherence method in vitro. Briefly, the human atrial appendage tissue was taken and the outer membrane and fat were removed. The tissue was then cut into small pieces of about 1 mm^3^ size. After being washed with PBS, the tissue samples were digested with 0.1% collagenase II solution (Gibco, Thermo Fisher Scientific, Inc.) in DMEM/F12 1:1 medium (HyClone) for 10 min. After that, samples were centrifuged and the supernatant was removed. After being digested and centrifuged once again, the resulting sediment was suspended in 10 mL of DMEM/F12 1:1 medium containing 20% fetal bovine serum (FBS; Gibco) and then pipetted into a 10-cm culture dish. Cells were cultured at 37 °C in a humidified atmosphere containing 5% CO_2_ in DMEM/F12 1:1 medium supplemented with 20% FBS, penicillin (100 U/mL), streptomycin (100 g/mL), and amphotericin B (250 ng/mL). For the first 4 days, we avoided moving the culture dish a lot to avoid affecting the attachment of the tissue fragment. After 2 weeks of primary culture, cells were sub-cultured for the subsequent experiments.

For fibroblasts identification, the fibroblasts obtained above were immuno-fluorescence stained with vimentin, a specific marker for fibroblasts. Briefly, cells were fixed in 40 g/L paraformaldehyde (4%) for 5–10 min and washed with PBS for three times. After that, cells were blocked with 0.5% Triton× 100 at room temperature for 30 min and washed with PBS three times. After being blocked with 1% BSA at room temperature for 1 h, cells were incubated with anti-vimentin (Cell Signaling Technology Inc., Danvers, MA, USA) at 4 °C overnight, followed by incubation with the secondary antibody IgG (Cell Signaling Technology Inc.) at room temperature for 1 h. After being washed with PBS for three times, cells were incubated with DAPI at room temperature for 5 min and observed under a fluorescence microscope (Nikon Corporation, Tokyo, Japan). The control group used PBS instead of primary anti-vimentin.

### Plasmid construction and cell transfection

To overexpress PVT1, the full-length PVT1 cDNA fragments were cloned into the pcDNA 3.1 plasmid (Invitrogen, USA), generating pcDNA3.1-PVT1. An empty pcDNA3.1 vector was used as the control. The human atrial fibroblasts were transfected with the respective constructs using Lipofectamine™ 2000 (Invitrogen, USA), following the manufacturer’s instructions. To knockdown PVT1 and Sp1, small interfering RNA (siRNA)-PVT1 (si-PVT1) and si-Sp1 were designed and synthesized by GenePharma (Shanghai, China). A scramble siRNA was used as negative control (si-Ctrl). The human atrial fibroblasts were transfected with siRNAs using Lipofectamine™ RNAiMAX Transfection Reagent (Invitrogen), according to the manufacturer’s instructions.

The miRNA double-stranded mimics for miR-128-3p or miR-128-3p inhibitors were purchased from GenePharma (Shanghai, China). When cells grew to 80–90% confluence, miR-128-3p mimics or miR-128-3p inhibitors were transfected into the human atrial fibroblasts.

After transfection for 48 h, the human atrial fibroblasts were harvested for qRT-PCR analysis to examine knockdown or overexpression efficiency.

### Cell proliferation assay

The proliferation of the cells in each group was measured by Cell Counting Kit-8 kit (CCK-8; Beyotime, Shanghai, China) according to the manufacturer’s instructions. Fibroblasts at passages 4–7 were seeded at a density of 2 × 10^3^/well in 96-well plates. Then, fibroblasts were transfected with the designated plasmids and treated with 1 μM Ang II (Sigma-Aldrich, St. Louis, MO, USA). After 48 h of incubation, cells were then incubated with 10 μL CCK-8 solution in each well at 37 °C for an additional 2 h. The optical density (OD) values were recorded at 450 nm on a full wavelength microplate analyzer (Molecular Device).

### Enzyme-linked immunosorbent assay (ELISA)

The levels of TGF-β1 in cell culture supernatants from human atrial fibroblasts were measured with an ELISA kit (R&D Systems, USA) according to the manufacturer’s protocol.

### RNA immunoprecipitation (RIP)

RIP was performed to investigate whether PVT1 and miR-128-3p were in the same RNA-induced silencing complex (RISC) complex. Briefly, cell lysates of human atrial fibroblasts were incubated with human anti-Argonaute2 (Ago2) or anti-IgG (Millipore) overnight at 4 °C. Normal IgG was used as a negative control. RNA-protein complexes were immunoprecipitated with protein A agarose beads and RNA was extracted by using TRIzol (Invitrogen). The resulting purified RNA was subjected to qRT-PCR analysis.

### Luciferase activity assay

The fragments of PVT1 and 3′-untranslated region (UTR) of Sp1 containing the predicted wild-type (wt) binding sites of miR-128-3p or mutated miR-128-3p binding sites (mut) were amplified by PCR and inserted into a pGL3 basic vector (Promega, Fitchburg, WI, USA) to generate PVT1-wt, PVT1-mut, Sp1-wt, and Sp1-mut. The constructed luciferase reporter vectors, miR-128-5p mimic or miR-negative control (mimic NC), as well as pRL-TK (expressing *Renilla* luciferase as the internal control), were co-transfected into human atrial fibroblasts. At 48 h post-transfection, luciferase activities were detected by a dual-luciferase reporter assay kit (Promega) and normalized to *Renilla* luciferase activity.

### Animal experiments

C57BL/6J mice (age of 6–7 weeks, weighing 20 ± 1 g) were randomly divided into four groups (*n* = 8/group): Control, Ang-II, Ang-II + si-Ctrl, and Ang-II-si-PVT1. Mice in the Ang-II, Ang-II + si-Ctrl, and Ang-II-si-PVT1 received an intraperitoneal injection of Ang II (1.5 μg/g/day; Sigma-Aldrich). Following 4 weeks of Ang-II injection, si-Ctrl or si-PVT1 were respectively injected into mice in the Ang-II + si-Ctrl and Ang-II-si-PVT1 groups via the tail vein. Following 2 weeks of siRNAs injection, mice were sacrificed and mouse cardiac function and hemodynamics related indicators were measured as listed in Table 2. The mouse atrial muscle tissues were harvested for the following histological examination. Western blot was performed as described above to determine the protein expression of collagen I, collagen III, TGF-β1, Smad2, and Smad3 in mouse atrial muscle tissues. The animal procedures in this study were approved by the Ethics Committee of the Renmin Hospital of Wuhan University.

**Table 2:** The effect of PVT1 knockdown on cardiac function and hemodynamics in mice (mean ± s)

Parameter	Control	Ang-II	Ang-II + si-ctrl	Ang-II + si-PVT1
Number	8	8	8	8
LVDd (mm)	3.58 ± 0.49	5.11 ± 0.86*	5.16 ± 0.76	4.05 ± 0.62^#^
LVDs (mm)	2.38 ± 0.46	4.77 ± 0.54*	4.64 ± 0.43	3.35 ± 0.84^#^
IVSd (mm)	0.63 ± 0.04	0.82 ± 0.03*	0.81 ± 0.04	0.71 ± 0.01^#^
IVSs (mm)	1.08 ± 0.02	1.23 ± 0.03*	1.22 ± 0.02	1.14 ± 0.02^#^
LVEF (%)	80.95 ± 2.15	51.23 ± 3.44*	52.04 ± 3.06	71.81 ± 2.95^#^
LVFS (%)	41.26 ± 3.68	25.75 ± 3.91*	25.48 ± 3.88	35.28 ± 3.84^#^

*LVDd* Left ventricular end diastolic diameter, *LVDs* left ventricular end systolic diameter, *IVSd* interventricular septal end-diastolic thinkness, *IVSs* interventricular septal end-systolic thickness, *LVEF* left ventricular ejection fraction, *LVFS* left ventricular fraction shortening. ^*^P < 0.05 vs. control, ^#^*P* < 0.05 vs. Ang-II + si-PVT1

### Histological examination

Heamatoxylin and eosin (H&E) staining was performed to evaluate the morphological changes of atrial muscle tissues. Masson’s trichrome staining was performed to evaluate the degree of atrial fibrosis. Briefly, the mouse atrial muscle tissues were fixed in 10% formaldehyde solution for 30 min. The paraffin-embedded tissues were then sectioned at 4-μm, and subjected to H&E staining and Masson’s trichrome staining following the routine procedures. The sections were analyzed under an Olympus BH-2 light microscope (Olympus Corporation).

### Statistical analysis

Statistical analyses were performed using SPSS 16.0 statistical software (SPSS, Inc., Chicago, IL, USA). The unpaired Student’s *t*-test was used to analyze differences between the two groups. One-way ANOVA was employed for the comparison of data between groups. The Spearman test was used to evaluate correlations (Prism 5; Graph-Pad Software, La Jolla, CA, USA). *p* < 0.05 was considered to indicate a statistically significant difference.

## Results

### Comparison of general clinical data between SR and AF group

The general clinical data between the SR and the AF group were listed in Table 1. There was no significant difference between the two groups in age, sex, cardiac function classification, SBP, DBP, RAD, and LVEF. However, LAD in the AF group was significantly higher than that in the SR group.

### PVT1 is increased in AF patients and positively with collagen I and collagen III

**Fig. 1:**
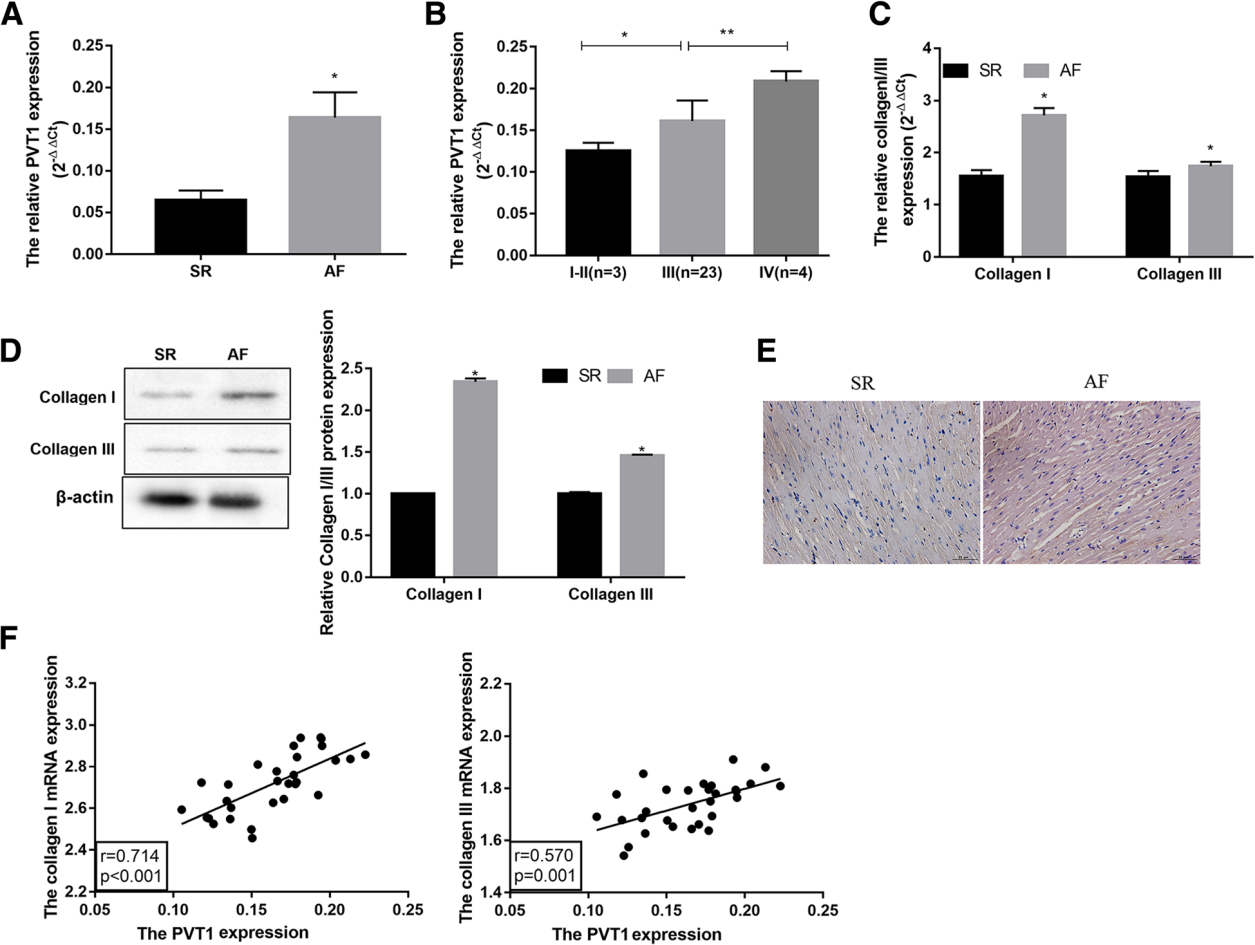
PVT1 is increased in AF patients and positively with collagen I and collagen II. The atrial muscle tissues were collected from SR (*n* = 20) and AF patients (*n* = 30) and subjected to the following experiments. **a** qRT-PCR analysis of PVT1 expression in human atrial muscle tissues. **b** qRT-PCR analysis of PVT1 expression from AF patients with different cardiac function classification (NYHA I-IV). PVT1 expression was gradually elevated with the increase of cardiac function classification. **c** qRT-PCR analysis of Collagen I and Collagen III mRNA levels in human atrial muscle tissues. **d** Western blot analysis of Collagen I and Collagen III protein levels in human atrial muscle tissues. β-actin served as the loading control. **e** Immunohistochemistry analysis of Collagen I showing the Collagen I-positive signal (brownish-yellow granules). Scale bar: 25 μm. **f** The positive correlation between PVT1 and Collagen I/III expression in human atrial muscle tissues from AF patients. **a**, **c**-**f** ^*^P < 0.05 vs. SR; **b** ^*^P < 0.05 and ^**^P < 0.01. Data are presented as mean ± SD. SR, sinus rhythm; AF, atrial fibrillation; PVT1, plasmacytoma variant translocation 1

PVT1 expression was notably up-regulated in human atrial muscle tissues in the AF group compared with the SR group (Fig. 1a). Furthermore, PVT1 expression was gradually elevated with the increase of cardiac function classification (Fig. 1b). Moreover, collagen I and collagen III, two of the main proteins in ECM, were significantly up-regulated in the AF group compared with the SR group, at both mRNA (Fig. 1c) and protein (Fig. 1d) levels. In addition, the immunohistological analysis further consolidated the upregulation of collagen I in atrial muscle tissues from the AF patients (Fig. 1e). Importantly, the results also showed that PVT1 was positively correlated with collagen I and collagen III in human atrial muscle tissues from AF patients (Fig. 1f). These data imply that increased PVT1 may play a potential role in regulating atrial fibrosis.

### Effect of PVT1 expression on Ang-II-induced fibroblasts proliferation, collagen production, and TGF-β1/Smad signaling activation

**Fig. 2:**
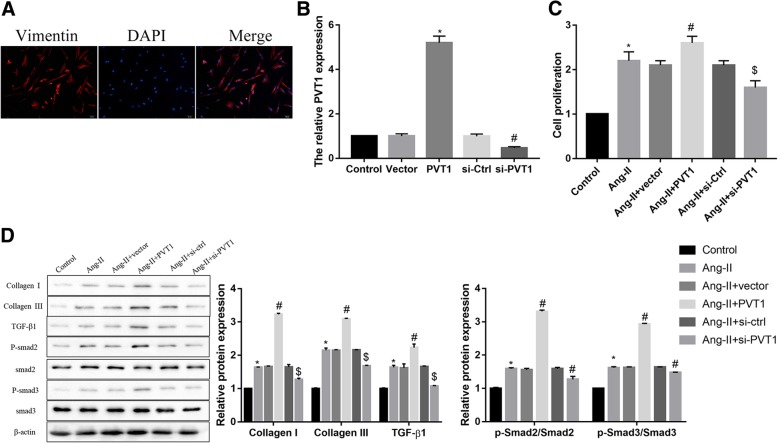
Effect of PVT1 expression on Ang-II-induced fibroblasts proliferation, collagen production, and TGF-β1/Smad signaling activation. **a** Identification of human atrial fibroblasts by vimentin immunostaining. Red stain is vimentin, a specific marker for fibroblasts. Blue stain is DAPI. **b** The overexpression and knockdown efficiency of PVT1 was confirmed by qRT-PCR. **c** Results of the CCK-8 assay showed that PVT1 overexpression facilitated the Ang-II-induced cell proliferation, whereas PVT1 knockdown exerted the opposite effect. **d** Western blot analysis showed that PVT1 overexpression further up-regulated the Ang-II-induced protein expression of Collagen I, Collagen II, TGF-β1/Smad signaling-related proteins, whereas PVT1 knockdown exerted the opposite effect. β-actin served as the loading control. **b** ^*^P < 0.05 vs. Vector, ^#^*P* < 0.05 vs. si-Ctrl; **c**-**d** ^*^P < 0.05 vs. Control, ^#^*P* < 0.05 vs. Ang-II + vector, ^$^*P* < 0.05 vs. Ang-II + si-Ctrl. Data are presented as mean ± SD. PVT1, plasmacytoma variant translocation 1; TGF-β1, transforming growth factor-β1

To explore the role of PVT1 in regulating atrial fibrosis, we isolated human atrial fibroblasts and transfected cells with pcDNA3.1-PVT1 and si-PVT1 to overexpress and silence PVT1 respectively, under Ang-II stimulation. The isolated fibroblasts were vimentin-positive with a purity of greater than 95%. The cytoplasm of fibroblasts stained with anti-vimentin in red showed filamentous at the edge of the nucleus stained with DAPI in blue, confirming the cells were atrial fibroblasts (Fig. 2a). Furthermore, qRT-PCR analysis confirmed the overexpression and knockdown efficiency of PVT1 in atrial fibroblasts (Fig. 2b). Importantly, Ang-II stimulation significantly promoted cell proliferation, which was then facilitated by PVT1 overexpression but blocked by PVT1 knockdown (Fig. 2c). Moreover, PVT1 overexpression further up-regulated the Ang-II-induced protein expression of collagen I, collagen II, TGF-β1/Smad signaling-related proteins, whereas PVT1 knockdown exerted the opposite effect (Fig. 2d). Similar secretion pattern of TGF-β1 was further consolidated by the data of ELISA analysis (Additional file 1: Figure S1). These data demonstrated that the Ang-II-induced fibroblasts proliferation, collagen production, and TGF-β1/Smad signaling activation was facilitated by PVT1 overexpression, but attenuated by PVT1 knockdown.

### PVT1 acts as s sponge for miR-128-3p to facilitate Sp1 expression

**Fig. 3:**
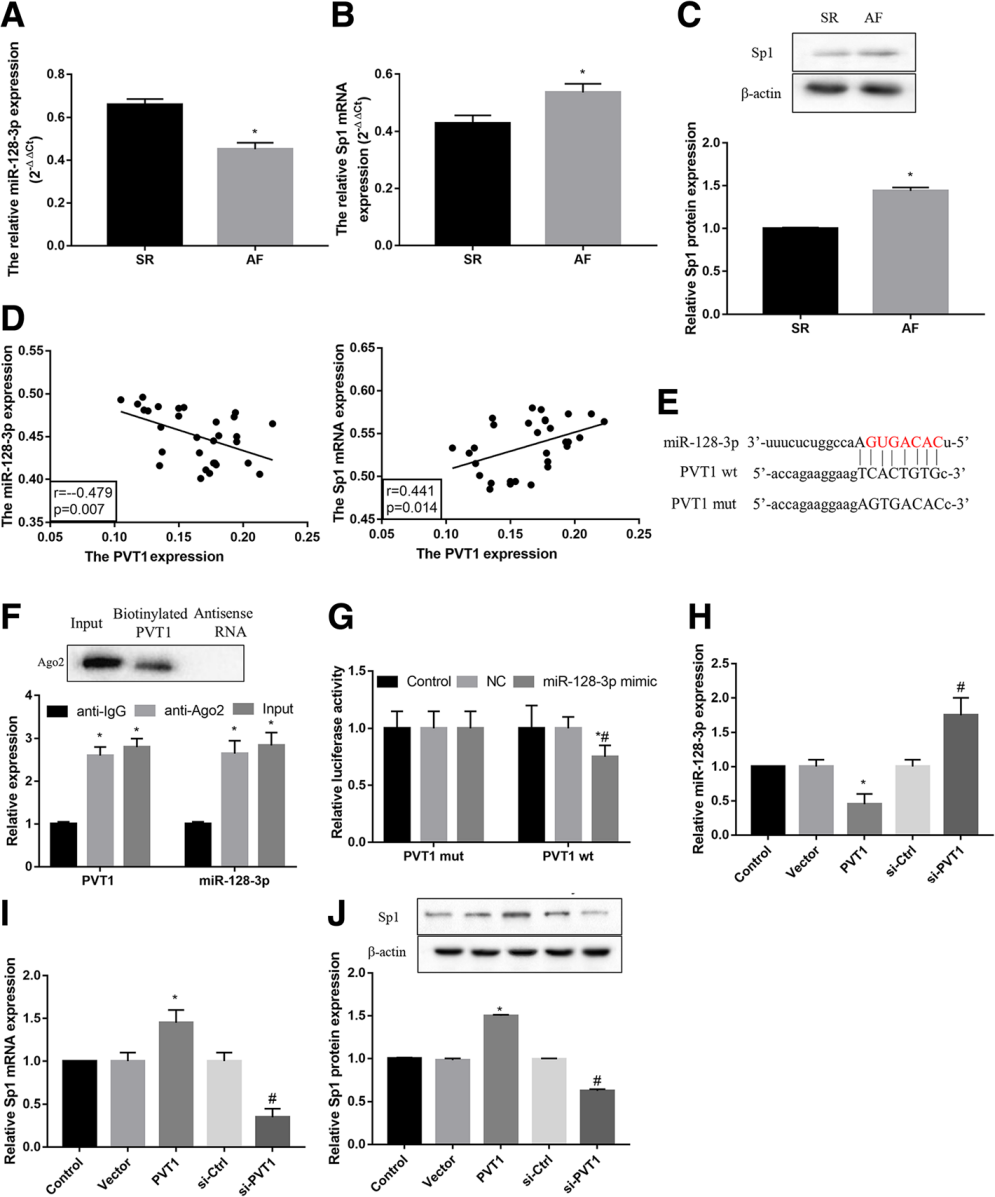
PVT1 acts as s sponge for miR-128-3p to facilitate Sp1 expression. **a** Compared with the SR group, the AF group showed significantly decreased expression of miR-128-3p (**a**) and increased mRNA (**b**) and protein (**c**) expression of Sp1 in human atrial muscle tissues. β-actin served as the loading control. **d** PVT1 expression was negatively correlated with miR-128-3p expression, whereas positively correlated with Sp1 mRNA expression in human atrial muscle tissues from AF patients. **e** The predicted binding sites between PVT1 and miR-128-3p (DIANA TOOLS-LncBase v.2). **f** Relative PVT1 and miR-128-3p expression presented as fold enrichment in Ago2 relative to normal IgG immunoprecipitates. RIP assays disclosed that PVT1 and miR-128-3p expressions were substantially enriched by Ago2 antibody compared with control IgG antibody. **g** Luciferase activity was measured in fibroblasts co-transfected with mimic NC or miR-128-3p mimic and PVT1-wt or PVT1-mut reporter at 48 h after transfection. PVT1 interacted directly with miR-128-3p. In addition, PVT1 overexpression inhibited miR-128-3p expression (**h**), elevated mRNA (**i**) and protein (**j**) levels of Sp1 in fibroblasts, whereas PVT1 knockdown exerted the opposite effect. **a**-**c** ^*^P < 0.05 vs. SR; (**f**) ^*^P < 0.05 vs. anti-IgG; (**g**) ^*^P < 0.05 vs. PVT1 wt + mimic NC, ^#^*P* < 0.05 vs. PVT1 wt + control; **h**-**j** ^*^P < 0.05 vs. vector, ^#^*P* < 0.05 vs. si-Ctrl. Data are presented as mean ± SD. SR, sinus rhythm; AF, atrial fibrillation; PVT1, plasmacytoma variant translocation 1; Sp1, specificity protein 1

To gain further insight into the molecular mechanism of PVT1 in regulating atrial fibrosis, we investigated the interaction among PVT1, miR-128-3p, and Sp1. Compared with the SR group, the AF group showed significantly decreased expression of miR-128-3p (Fig. 3a) and increased mRNA (Fig. 3b) and protein (Fig. 3c) expression of Sp1 in atrial muscle tissues. Furthermore, PVT1 expression was negatively correlated with miR-128-3p but positively correlated with Sp1 in atrial muscle tissues from AF patients (Fig. 3d).

Intriguingly, our bioinformatics analysis (DIANA TOOLS) revealed that PVT1 harbors putative binding sites of miR-128-3p (Fig. 3e), Moreover, RIP assays disclosed that PVT1 and miR-128-3p expressions were substantially enriched by Ago2 antibody compared with control IgG antibody (Fig. 3f). Luciferase activity assay showed that miR-128-3p mimic led to a notable decrease in luciferase activity in PVT1-WT reporter compared with the mimic NC group, whereas had no obvious effect on luciferase activity in PVT1-MUT reporter (Fig. 3g). Together, these results verified PVT1 interacted directly with miR-128-3p.

In addition, PVT1 overexpression significantly inhibited miR-128-3p expression (Fig. 3h), whereas induced Sp1 expression at both mRNA (Fig. 3i) and protein (Fig. 3j) levels. In contrast, PVT1 knockdown exerted the opposite effects. Collectively, these data suggest that PVT1 acts as a sponge for miR-128-3p to facilitate Sp1 expression.

### PVT1 facilitates the Ang-II-induced fibroblasts proliferation, collagen production, and TGF-β1/Smad signaling activation via miR-128-3p/Sp1 axis

**Fig. 4:**
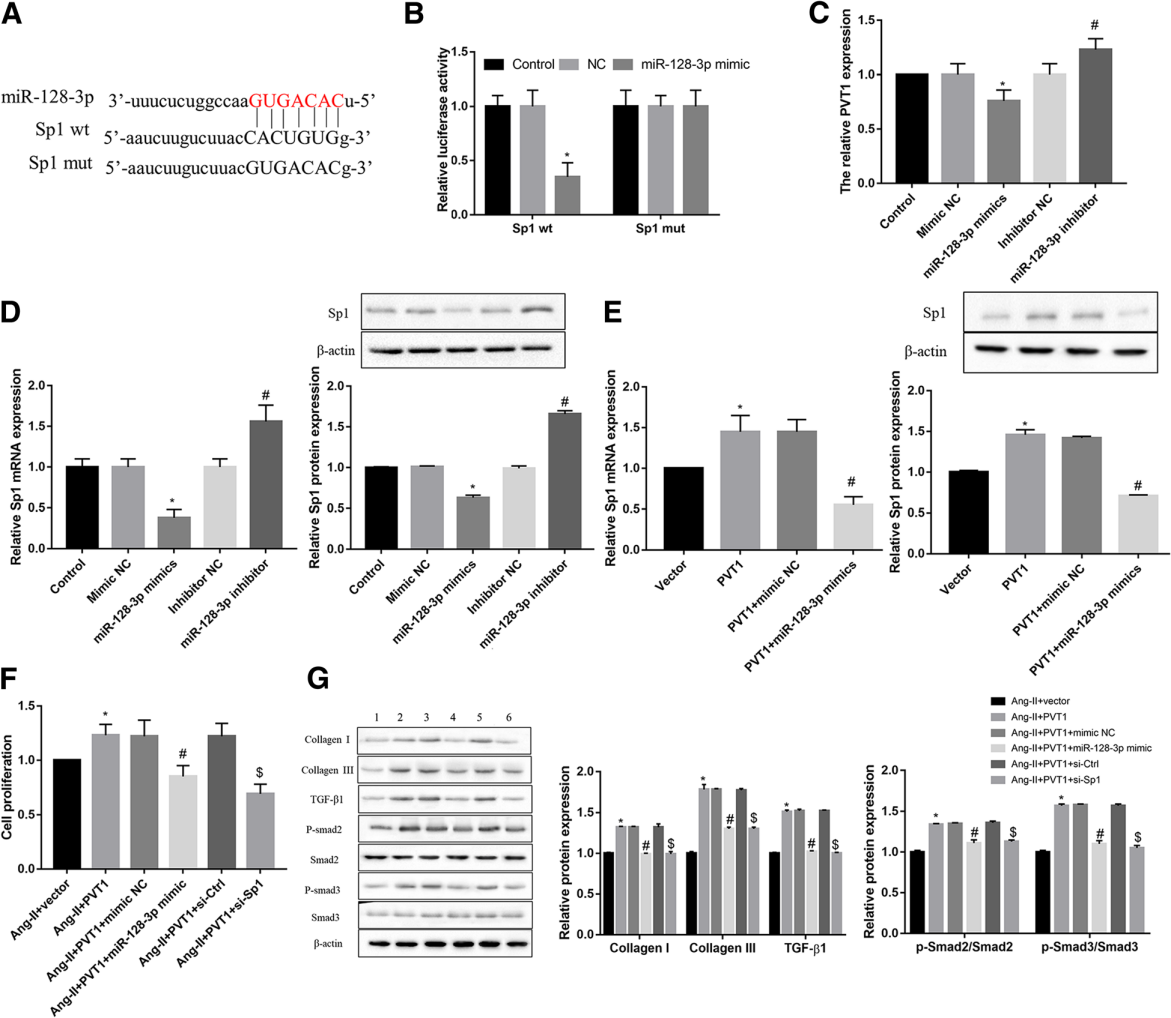
PVT1 promotes atrial fibrosis by regulating the TGF-β1/Smad signaling pathway via the miR-128-3p/Sp1 axis. **a** The predicted binding site between miR-128-3p and 3′-UTR of Sp1 (Targetscan). **b** Luciferase assay confirmed that Sp1 was a target of miR-128-3p. miR-128-3p mimic significantly suppressed PVT1 expression (**c**), and the mRNA and protein expression of Sp1 (**d**). miR-128-3p inhibitor exerted the opposite effect. **e** miR-128-3p mimic significantly reversed the PVT1 overexpression-induced Sp1 expression, at both mRNA and protein levels. Effect of miR-128-3p mimic and Sp1 silencing on the PVT1 overexpression-induced fibroblasts proliferation (**f**), collagen production, and TGF-β1/Smad signaling activation (**g**) was shown. **b** ^*^P < 0.05 vs. Sp1 wt + mimic NC; (C-D) ^*^P < 0.05 vs. mimic NC, ^#^*P* < 0.05 vs. inhibitor NC; (**e**) ^*^P < 0.05 vs. vector, ^#^*P* < 0.05 vs. PVT1 + mimic NC; (**f**-**g**) ^*^P < 0.05 vs. Ang-II + vector, ^#^*P* < 0.05 vs. Ang-II + PVT1 + mimic NC, ^$^*P* < 0.05 vs. Ang-II + PVT1 + si-Ctrl. Data are presented as mean ± SD. Sp1, specificity protein 1; PVT1, plasmacytoma variant translocation 1; TGF-β1, transforming growth factor-β1

We next clarified whether the miR-128-3p/Sp1 axis was responsible for PVT1-mediated facilitation of fibroblasts proliferation, collagen production, and TGF-β1/Smad signaling activation under Ang-II stimulation. As shown in Fig. 4a and b, the luciferase assay confirmed that Sp1 was a target of miR-128-3p. Furthermore, miR-128-3p mimic significantly suppressed PVT1 expression (Fig. 4c), and the mRNA and protein expression of Sp1 (Fig. 4d). In contrast, miR-128-3p inhibitor exerted the opposite effect (Fig. 4c and d). Moreover, miR-128-3p mimic significantly reversed the PVT1 overexpression-induced Sp1 expression, at both mRNA and protein levels (Fig. 4e). More importantly, both miR-128-3p mimic and Sp1 silencing significantly reversed the PVT1 overexpression-mediated facilitation of fibroblasts proliferation (Fig. 4f), collagen production, and TGF-β1/Smad signaling activation under Ang-II stimulation (Fig. 4g). Similar secretion pattern of TGF-β1 was further consolidated by the data of ELISA analysis (Additional file 2: Figure S2). These results indicate PVT1 facilitates the Ang-II-induced fibroblasts proliferation, collagen production, and TGF-β1/Smad signaling activation via miR-128-3p/Sp1 axis.

### PVT1 knockdown attenuates the Ang-II-induced atrial fibrosis

To further in vivo verify the role of PVT1 in atrial fibrosis in mice, Ang-II was injected into mice to induce cardiac fibrosis for 4 weeks, and si-PVT1 or si-Ctrl (as control) was subsequently injected into Ang-II-treated mice. Following 2 weeks of siRNAs injection, the knockdown efficiency of PVT1 in atrial muscle tissues was confirmed by qRT-PCR (Additional file 3: Figure S3). The mouse cardiac function and hemodynamics-related indicators were measured as listed in Table 2. Compared with the mice in the control group, the mice in the Ang-II group showed significantly higher levels of LVDd, LVDs, IVSd, and IVSs, but lower levels of LVEF and LVFS. Importantly, compared with the mice in the Ang-II + si-Ctrl group, the mice in the Ang-II + si-PVT1 group exhibited a significant decrease in levels of LVDd, LVDs, IVSd, and IVSs, but a significant increase in LVEF and LVFS. These results indicate that PVT1 knockdown attenuates the Ang-II-induced dysfunction of mouse cardiac function and hemodynamics.

**Fig. 5:**
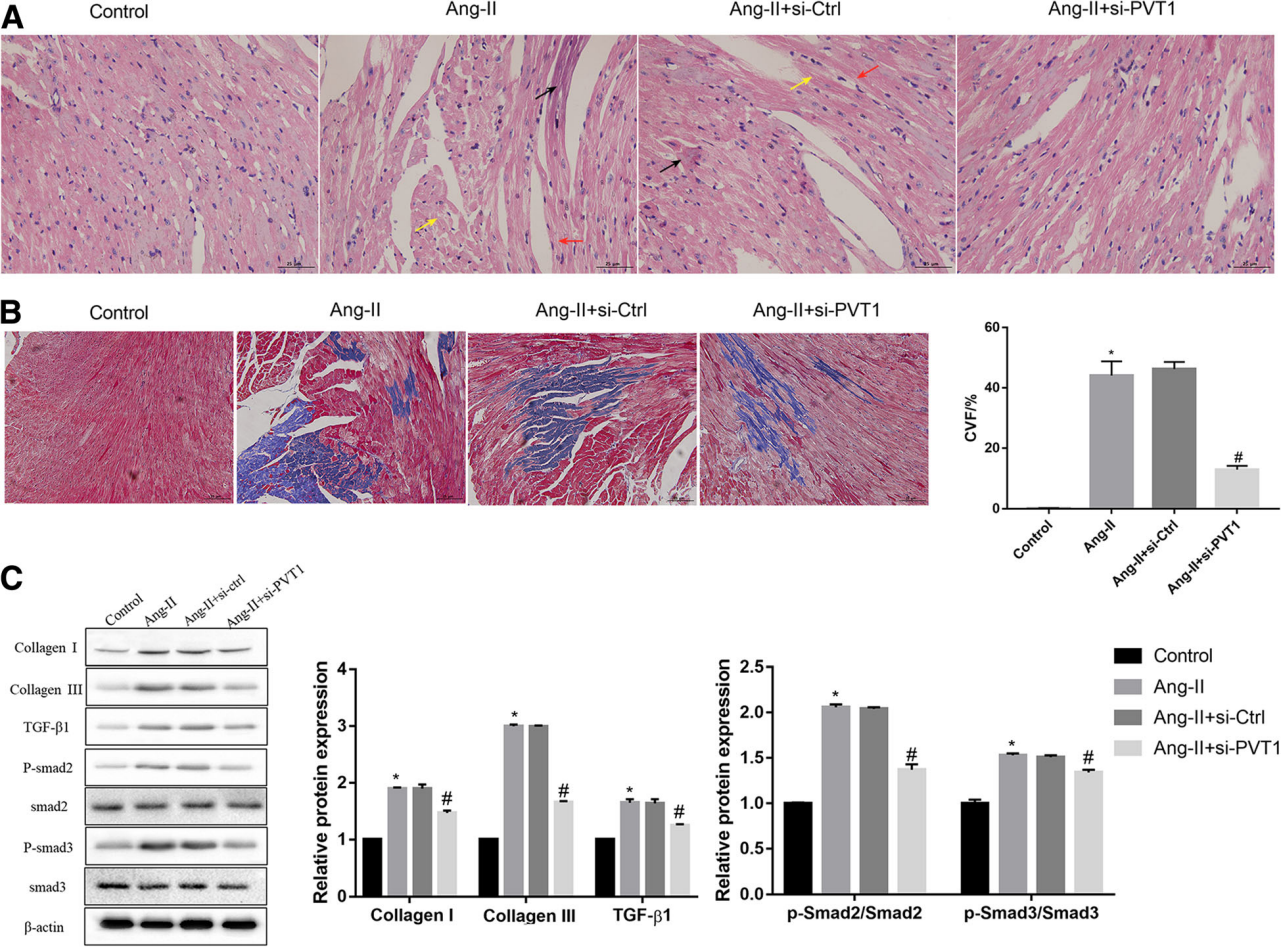
PVT1 knockdown attenuates the Ang-II-induced atrial fibrosis. Ang-II was injected into mice to induce cardiac fibrosis for 4 weeks, and si-PVT1 or si-Ctrl (as control) was subsequently injected into Ang-II-treated mice. Following 2 weeks of siRNAs injection, (**a**) HE staining was performed to evaluate the morphological changes of mouse atrial muscle tissues. Scale bar: 25 μm. Black arrows indicate increased myocardial interstitial fibrosis; red arrows indicate expanded nuclear spacing; yellow arrows indicate myocardial fiber disarray. **b** Masson staining was performed to evaluate the degree of atrial fibrosis. Fibrotic tissue was stained in blue. Collagen volume fraction (CVF, %) was calculated. Scale bar: 25 μm. **c** Western blot was performed to determine the protein expression of Collagen I, Collagen III, TGF-β1, Smad2 and Smad3 in mouse atrial muscle tissues. β-actin served as the loading control. **c** ^*^P < 0.05 vs. control, ^#^*P* < 0.05 vs. Ang-II + si-Ctrl. *N* = 8 mice in each group. Data are presented as mean ± SD. PVT1, plasmacytoma variant translocation 1; TGF-β1, transforming growth factor-β1

In addition, compared with the mice in the control group, the mice in the Ang-II group showed myocardial fiber disarray, expanded nuclear spacing, and markedly elevated atrial fibrosis (Fig. 5a and b). Moreover, the mice in the Ang-II group also showed significantly higher protein expression of collagen I, collagen III, TGF-β1, p-Smad2, and p-Smad3 in atrial muscle tissues (Fig. 5c). Importantly, PVT1 knockdown effectively attenuated the Ang-II-induced inflammatory infiltration and atrial fibrosis (Fig. 5a and b), and mitigated the Ang-II-induced collagen production and TGF-β1/Smad signaling activation (Fig. 5c).

## Discussion

Several studies have shown that lncRNAs are aberrantly expressed in AF patients and implicated in AF development. For instance, lncRNA AK055347 is upregulated in AF patients and regulates mitochondrial energy production in myocardiocytes (Chen et al. 2016). A more recent study revealed that lncRNA KCNQ1 overlapping transcript 1 (KCNQ1OT1) is upregulated in Ang-II-induced AF mice hearts and promotes Ang-II-induced AF by acting as a sponge for miR-384b to facilitate CACNA1C expression (Shen et al. 2018). Nevertheless, the exact role of lncRNAs in AF still need to be further elucidated. In this study, we observed upregulated lncRNA PVT1 in atrial muscle tissues from AF patients and positively with collagen I and collagen III. Collagen I and collagen III are two of the main proteins in ECM. ECM production contributes to atrial fibrosis. Thus, these results indicated the potential role of PVT1 in AF.

Atrial fibrosis is the hallmark of structural remodeling in AF and associated with AF recurrences, resistance to therapy and complications (Nattel 2017; Dzeshka et al. 2015). Atrial fibrosis is characterized by abnormal proliferation of atrial fibroblasts and excessive deposition of ECM (Wang et al. 2017). Here, in Ang-II-treated human atrial fibroblasts, PVT1 overexpression facilitated the Ang-II-induced fibroblasts proliferation and collagen production, whereas PVT1 knockdown exerted the opposite effect. Our in vivo assay further confirmed that PVT1 knockdown attenuated the Ang-II-induced mouse atrial fibrosis. All these results indicated the pro-fibrotic role of PVT1 in AF.

TGF-β1/Smad pathway plays an important role in matrix remodeling, capable of activating atrial fibroblasts differentiation and promoting cellular migration, proliferation and ECM synthesis, which are associated with atrial fibrosis and AF (Guo et al. 2018; Li et al. 2017; Tao et al. 2018). Several studies have demonstrated that TGF-β1 had a profibrotic effect via Smad proteins, which could potentiate atrial fibrosis and AF (Guo et al. 2018). Previous in vitro studies have identified that TGF-β1 increased expression of p-Smad2 and collagen content, and converted rat atrial fibroblasts into myofibroblasts, which induced the atrial fibrosis phenotype (Zhang et al. 2013; Yeh et al. 2013). Of note, the results in this study revealed that PVT1 knockdown suppressed TGF-β1/Smad signaling, both in vitro and in vivo. This implied the involvement of TGF-β1/Smad signaling in the PVT1-mediated promotion of fibrosis.

TGF-β1 can bind to Sp1 and can be activated by the transcriptional factor Sp1 (Martin-Gallausiaux et al. 2018). A previous study showed that miR-29b inhibits endometrial fibrosis by blockade of the Sp1-TGF-β1/Smad pathway in a rat model (Li et al. 2016), indicating the role of Sp1-TGF-β1/Smad signaling in regulating fibrosis. LncRNAs can exert certain roles by acting as a ceRNA to segregate miRNAs away from target mRNAs (Xiong et al. 2018; Wei et al. 2018). For example, PVT1 facilitates HIF-1α expression through acting as ceRNA for miR-199a-5p in non-small cell lung cancer (Han et al. 2019). These findings further encouraged us to investigate whether PVT1 regulated Sp1 expression and whether ceRNA mechanisms involved in this process. Our bioinformatics analysis showed a possible PVT1-miR-128-3p-Sp1 axis and our experiments confirmed that PVT1 acted as a sponge for miR-128-3p to facilitate Sp1 expression. Importantly, both miR-128-3p mimic and Sp1 silencing significantly abrogated the PVT1 overexpression-mediated facilitation of fibroblasts proliferation, collagen production, and TGF-β1/Smad signaling activation in Ang-II-treated fibroblasts. Our results revealed a ceRNA mechanism underlying the pro-fibrotic role of PVT1 in AF. Consistent with our partial results, PVT1 has been found to act as a sponge of miR-128-3p to facilitate the targets of miR-128 (Fu et al. 2018; Yu et al. 2019). In addition, Sp1 has been confirmed as a target of miR-128 in bovine skeletal muscle satellite cells (Dai et al. 2016).

## Conclusions

In conclusion, lncRNA PVT1 promoted fibroblasts proliferation, collagen production, and mice atrial fibrosis via the miR-128-3p-Sp1-TGF-β1-Smad axis. Our results indicate that PVT1 may serve as a novel therapeutic target for therapy for AF.

## Additional files

Additional file 1: **Figure S1.** ELISA analysis showed that PVT1 overexpression further up-regulated the Ang-II-induced secretion of TGF-β1, whereas PVT1 knockdown exerted the opposite effect. ^*^P < 0.05 vs. Control, ^#^*P* < 0.05 vs. Ang-II + vector, ^$^*P* < 0.05 vs. Ang-II + si-Ctrl. Data are presented as mean ± SD. PVT1, plasmacytoma variant translocation 1; TGF-β1, transforming growth factor-β1. (TIF 76 kb)

Additional file 2: **Figure S2.** Effect of miR-128-3p mimic and Sp1 silencing on the PVT1 overexpression-induced secretion of TGF-β1. Levels of TGF-β1 were measured using ELISA. ^*^P < 0.05 vs. Ang-II + vector, ^#^*P* < 0.05 vs. Ang-II + PVT1 + mimic NC, ^$^*P* < 0.05 vs. Ang-II + PVT1 + si-Ctrl. Data are presented as mean ± SD. Sp1, specificity protein 1; PVT1, plasmacytoma variant translocation 1; TGF-β1, transforming growth factor-β1. (TIF 98 kb)

Additional file 3: **Figure S3.** Ang-II was injected into mice to induce cardiac fibrosis for 4 weeks, and si-PVT1 or si-Ctrl (as control) was subsequently injected into Ang-II-treated mice. Following 2 weeks of siRNAs injection, the knockdown efficiency of PVT1 in atrial muscle tissues was confirmed by qRT-PCR. ^*^P < 0.05 vs. control, ^#^*P* < 0.05 vs. Ang-II + si-Ctrl. *N* = 8 mice in each group. Data are presented as mean ± SD. PVT1, plasmacytoma variant translocation 1. (TIF 114 kb)

## Abbreviations

AF: Atrial fibrillation
Ang-II: Angiotensin-II
ceRNA: Competitive endogenous RNA
ECM: Extracellular matrix
H&E: Heamatoxylin and eosin
IHC: Immunohistochemistry
lncRNAs: long non-coding RNAs
LVEF: Left ventricular ejection fraction
PVT1: Plasmacytoma variant translocation 1
RIP: RNA immunoprecipitation
RISC: RNA-induced silencing complex
Sp1: Specificity protein 1
SR: Sinus rhythm
TGF-β1: Transforming growth factor beta 1
UTR: Untranslated region
wt: wild-type
