# Supplementary figures and images for: LncRNA PVT1 regulates atrial fibrosis via miR-128-3p-SP1-TGF-β1-Smad axis in atrial fibrillation
Source: Mol Med. 2019 Mar 20;25:7. doi: 10.1186/s10020-019-0074-5 (PMC6425687; doi:10.1186/s10020-019-0074-5)

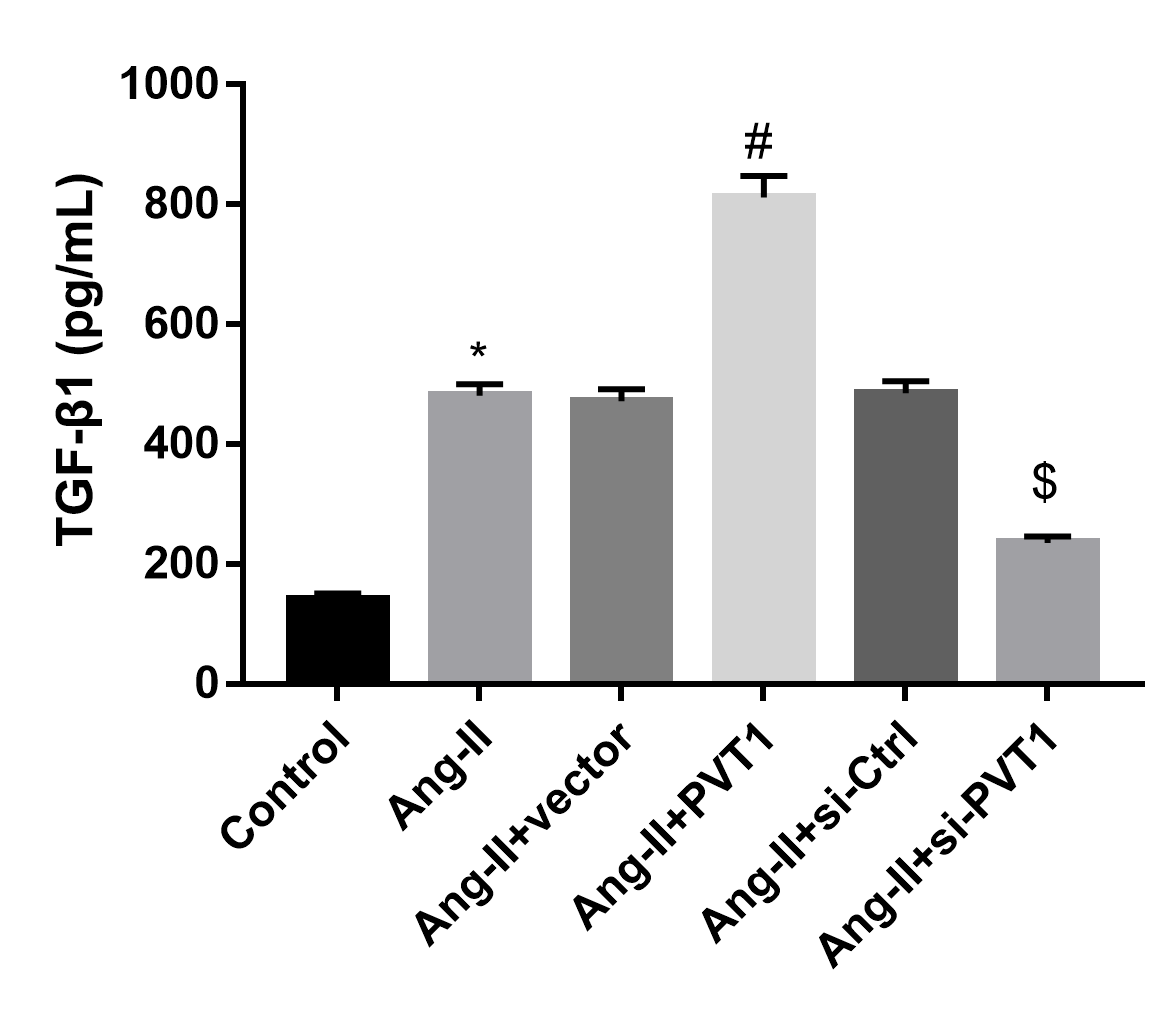

Supplement: Supplementary file 1 — Figure S1. ELISA analysis showed that PVT1 overexpression further up-regulated the Ang-II-induced secretion of TGF-β1, whereas PVT1 knockdown exerted the opposite effect. *P < 0.05 vs. Control, #P < 0.05 vs. Ang-II + vector, $P < 0.05 vs. Ang-II + si-Ctrl. Data are presented as mean ± SD. PVT1, plasmacytoma variant translocation 1; TGF-β1, transforming growth factor-β1. (TIF 76 kb) [file 10020_2019_74_MOESM1_ESM.tif]

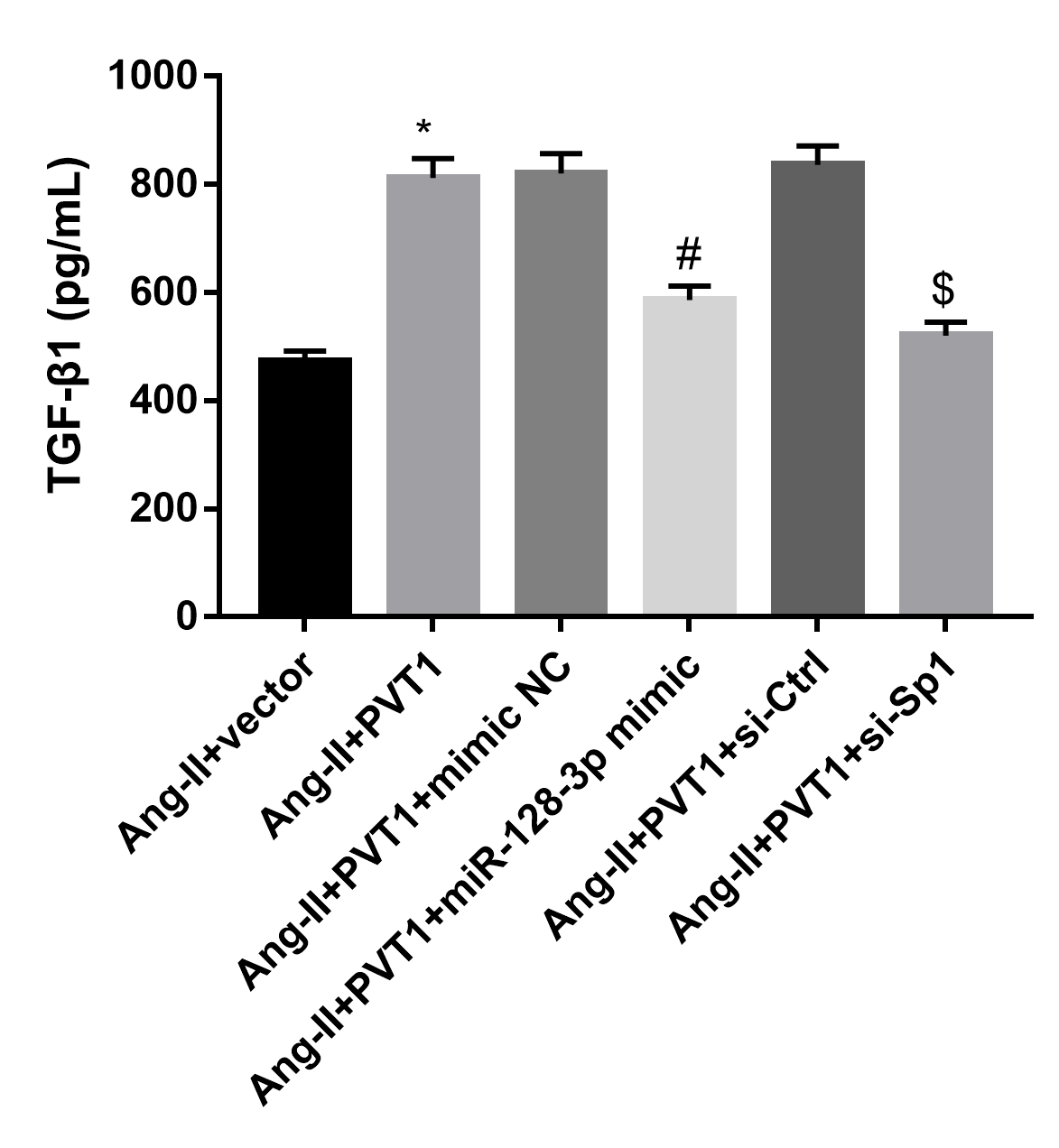

Supplement: Supplementary file 2 — Figure S2. Effect of miR-128-3p mimic and Sp1 silencing on the PVT1 overexpression-induced secretion of TGF-β1. Levels of TGF-β1 were measured using ELISA. *P < 0.05 vs. Ang-II + vector, #P < 0.05 vs. Ang-II + PVT1 + mimic NC, $P < 0.05 vs. Ang-II + PVT1 + si-Ctrl. Data are presented as mean ± SD. Sp1, specificity protein 1; PVT1, plasmacytoma variant translocation 1; TGF-β1, transforming growth factor-β1. (TIF 98 kb) [file 10020_2019_74_MOESM2_ESM.tif]

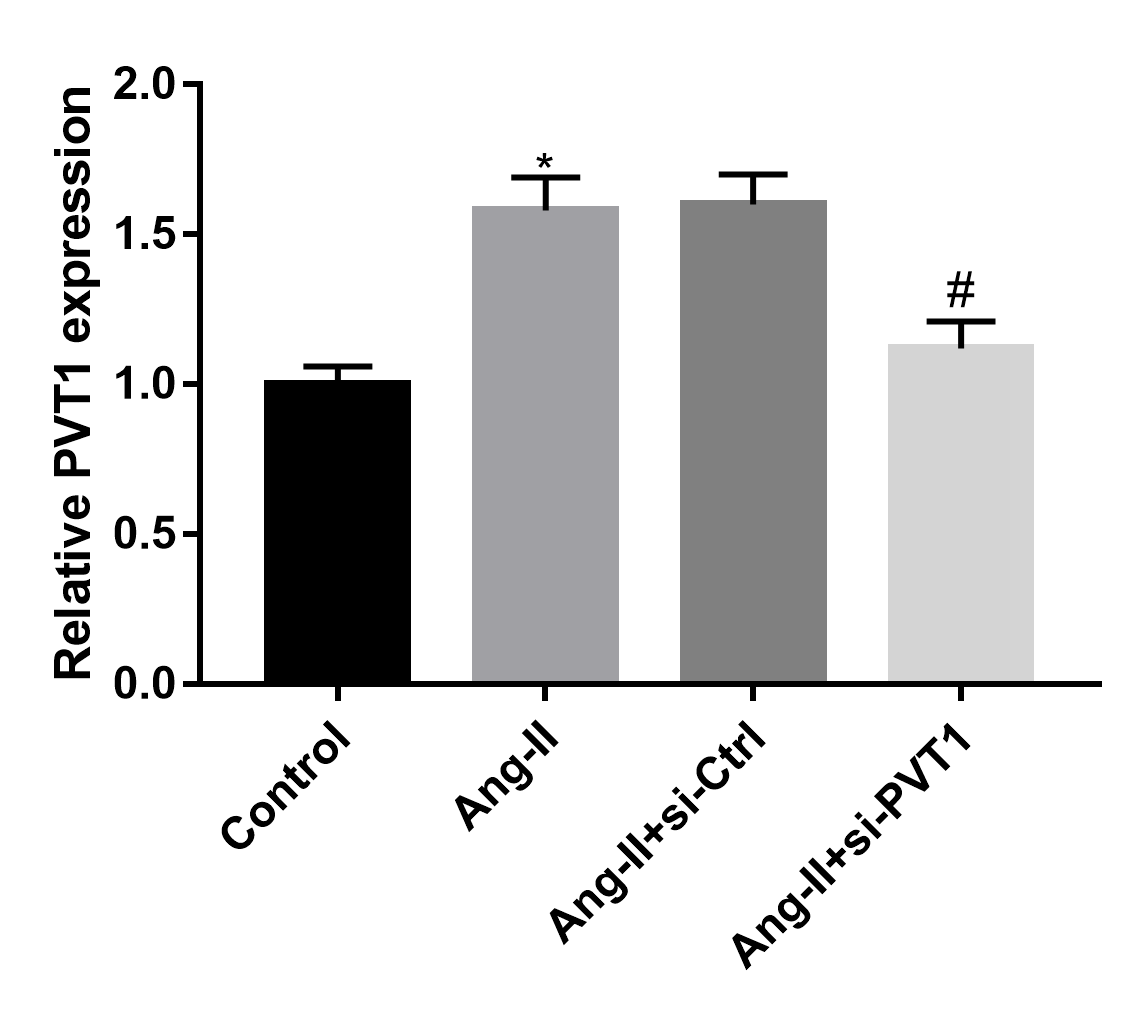

Supplement: Supplementary file 3 — Figure S3. Ang-II was injected into mice to induce cardiac fibrosis for 4 weeks, and si-PVT1 or si-Ctrl (as control) was subsequently injected into Ang-II-treated mice. Following 2 weeks of siRNAs injection, the knockdown efficiency of PVT1 in atrial muscle tissues was confirmed by qRT-PCR. *P < 0.05 vs. control, #P < 0.05 vs. Ang-II + si-Ctrl. N = 8 mice in each group. Data are presented as mean ± SD. PVT1, plasmacytoma variant translocation 1. (TIF 114 kb) [file 10020_2019_74_MOESM3_ESM.tif]
